# The design of the SAFE or SORRY? study: a cluster randomised trial on the development and testing of an evidence based inpatient safety program for the prevention of adverse events

**DOI:** 10.1186/1472-6963-9-58

**Published:** 2009-04-01

**Authors:** Betsie GI van Gaal, Lisette Schoonhoven, Marlies EJL Hulscher, Joke AJ Mintjes, George F Borm, Raymond TCM Koopmans, Theo van Achterberg

**Affiliations:** 1 IQ healthcare, Radboud University Nijmegen Medical Centre, Nijmegen, the Netherlands; 2 Faculty of Health and Social Studies, HAN University of Applied Sciences, Nijmegen, the Netherlands; 3 Department of Epidemiology, Biostatistics and HTA, Radboud University Nijmegen Medical Centre, Nijmegen, the Netherlands; 4 Department of Primary and Community Care, Centre for family Medicine, Geriatric Care and Public Health Medicine, Radboud University Nijmegen Medical Centre, Nijmegen, the Netherlands

## Abstract

**Background:**

Patients in hospitals and nursing homes are at risk of the development of, often preventable, adverse events (AEs), which threaten patient safety. Guidelines for prevention of many types of AEs are available, however, compliance with these guidelines appears to be lacking. Besides general barriers that inhibit implementation, this non-compliance is associated with the large number of guidelines competing for attention. As implementation of a guideline is time-consuming, it is difficult for organisations to implement all available guidelines. Another problem is lack of feedback about performance using quality indicators of guideline based care and lack of a recognisable, unambiguous system for implementation. A program that allows organisations to implement multiple guidelines simultaneously may facilitate guideline use and thus improve patient safety.

The aim of this study is to develop and test such an integral patient safety program that addresses several AEs simultaneously in hospitals and nursing homes. This paper reports the design of this study.

**Methods and design:**

The patient safety program addresses three AEs: pressure ulcers, falls and urinary tract infections. It consists of bundles and outcome and process indicators based on the existing evidence based guidelines. In addition it includes a multifaceted tailored implementation strategy: education, patient involvement, and a computerized registration and feedback system. The patient safety program was tested in a cluster randomised trial on ten hospital wards and ten nursing home wards. The baseline period was three months followed by the implementation of the patient safety program for fourteen months. Subsequently the follow-up period was nine months. Primary outcome measure was the incidence of AEs on every ward. Secondary outcome measures were the utilization of preventive interventions and the knowledge of nurses regarding the three topics. Randomisation took place on ward level. The results will be analysed separately for hospitals and nursing homes.

**Discussion:**

Major challenges were the development of the patient safety program including a digital registration and feedback system and the implementation of the patient safety program.

**Trial registration:**

Trial registration: ClinicalTrials.gov ID [NCT00365430]

## Background

Over the past seventeen years several studies showed that patients are at risk of injuries or even death as a result of care delivered in hospitals [1-11]. These studies show that 2.9 to 16.6% of patients in acute care hospitals experienced at least one adverse event (AE) (Table 1) [1,2,9-11]. In 5 to 13% of these events the patients died [1-3,7,9-11]. Half of all events are considered preventable [1,3,6,9-11]. While these studies did not include nursing homes, other studies show that AEs, such as urinary tract infection, pneumonia, falls, pressure ulcers and medication errors, also occur frequently in nursing homes [12-14]. These events can often be linked directly to suboptimal nursing care, and they are generally considered preventable.

**Table 1:** Definitions

**Adverse event**
An adverse event (AE) is defined as an unintended injury that results in prolonged stay, disability at the time of discharge, or death and is caused by health care management rather than by the patient's underlying disease process [1,3,9,11].
**Bundle**
A bundle is a structured way of improving the processes of care and patient outcomes: a small, straightforward set of practices – generally three to five – that, when performed collectively and reliably, have been proven to improve patient outcomes [17].

Many guidelines for the improvement of nursing care are available, however compliance with these guidelines appears to be lacking [15]. Generally, many factors or barriers may influence compliance -or noncompliance- with a guideline. These general barriers may be related to the individual (e.g. knowledge, skills, attitudes, motivation) or the individual's social context (e.g. patients, colleagues, culture), and the organisational setting (e.g. financial, equipment). Moreover, the large number of guidelines competing for attention makes it difficult to keep track of all of them. In addition organisations must translate each guideline to their own target group, and develop and organise their own information and education, which is a time-consuming process. Also, there is a lack of insight into actual performance of guideline based care, e.g. by using quality indicators [16]. As a result it is difficult to implement all available guidelines necessary for good quality daily nursing care. This situation is at odds with the responsibility of professionals to ensure patient safety. A program that allows organisations to implement multiple guidelines simultaneously may facilitate guideline use and thus improve patient safety.

The aim of this study is to develop and test such an integral patient safety program that addresses several AEs simultaneously in hospitals and nursing homes.

In this paper we will report on the design of this study, which has two phases. The first phase concerns the development of the patient safety program for three frequently occurring nursing care related AEs: pressure ulcers, falls and urinary tract infections. The second phase describes the evaluation of the patient safety program in a cluster randomised trial.

## Methods and design

### Phase 1: the development of the patient safety program

#### General focus of the program

From September 2005 – July 2006 we developed the integral patient safety program (SAFE or SORRY?) for the prevention of pressure ulcers, falls and urinary tract infections in hospitals and nursing homes. The program consists of bundles [17] (Table 1) and outcome and process indicators based on evidence based guidelines for pressure ulcers, falls and urinary tract infections.

For the implementation of guidelines, multifaceted implementation strategies are probably more effective than single strategies, as multifaceted strategies address multiple barriers to guideline adherence [16]. Therefore, we aimed at developing a multifaceted strategy for the implementation of these bundles.

#### Development

We developed the patient safety program with experts on each topic by collecting the existing guidelines [18-26] and supplementary material [27-40]. Based on this information the research group and the experts achieved consensus about the essence of the guidelines and formulated the bundles and indicators (Table 2). They developed a multifaceted implementation strategy consisting of education, patient involvement, feedback through a computerized registration program and an implementation plan for every ward (Table 3).

**Table 2:** Process (P) and outcome (O) indicators

**Pressure Ulcers**
% patients where nurses assessed pressure ulcer risk (P)
% patients at risk for pressure ulcers (O)
% patients with pressure ulcers grade 2 or worse (O; prevalence)
% patients developing nonblanchable erythema (O; incidence)
% patients developing pressure ulcers grade 2 or worse (O; incidence)
% patients developing pressure ulcers grade 2 or worse at the heels (O; incidence)
% at risk patients receiving permanent adequate preventive measures (P)
% patients developing pressure ulcers despite the preventive measures (O)
% patients with pressure ulcers increasing in grade and/or becoming more serious (O)
**Urinary tract infection**
% patients where nurses assessed risk for urinary tract infection (P)
% patients at risk for urinary tract infections (O)
% patients with urinary tract infections (O; prevalence)
% patients with fecal incontinence with urinary tract infections (O; prevalence)
% patients with urinary tract infections who have of had a bladder catheter (O; prevalence)
% patients developing urinary tract infections (O; incidence)
% at risk patients receiving permanent adequate preventive measures (P)
% patients with an appropriate/correct indication for indwelling bladder catheter (P)

**Falls**
% patients where nurses assessed risk for falling (P)
% patients at risk for falls (O)
% patient falls (O; prevalence)
% patients at risk that received multi-factorial measures (P)
% patients in which both risk factors and multi-factorial measures were evaluated regularly (P)
% patient that fell despite multi-factorial measures (O)

**Table 3:** Operational implementation strategies

**Education**
Group lesson on the wards for all nurses
A CDrom with education material and a knowledge test
Case discussions on every ward
**Patient involvement**
An information folders for the prevention of pressure ulcers, urinary tract infection and falls, separately. In addition to giving oral information nurses were asked to give the folder to patients at risk for the specific AE.

**Feedback**
The nurses register the patient's daily care and the presence or absence of an AE in a computerized registration system. This digital program generates feedback by charts on the process and outcome indicators.

#### Tailoring

We discussed the bundles and indicators with the user group. This group consisted of two researchers (LS and BGIvG), seventeen future users of the patient safety program, two medical doctors and an implementation expert (MEJLH) and met five times. During the first meeting everyone was informed about the aim and work methods. During the next three meetings the group was split up into two smaller groups: a group with users from the hospitals and a group with users from the nursing homes. In each group we had focus discussions about the use of the bundles and indicators and the expected barriers for implementation. During the fifth meeting the group tested the computerized registration program. With this information, and the outcome on the knowledge test from the baseline measurement (phase 2), we tailored the education for the nurses to each individual ward in the intervention group. In a last meeting, the users of the intervention group tested the final educational material and the patient information. In order not to contaminate the control group with the elaborated education material and patient information, the users of this group were not invited to this last meeting.

Table 3 describes the concrete implementation strategies for the patient safety program. In addition, every intervention ward appointed two key nurses to the study. Together with the head nurse they were responsible for the implementation of the patient safety program on their ward. At the start of the implementation period these key nurses received a training in the use of the patient safety program. We also discussed the results of the baseline measurements (phase 2) and the educational material, and all educational activities on the wards were planned and organised. The key nurses and the researcher had periodical contact about the progress on the ward, throughout the implementation period.

### Phase 2: cluster randomised clinical trial to evaluate the patient safety program

#### Study design and setting

**Figure 1:**
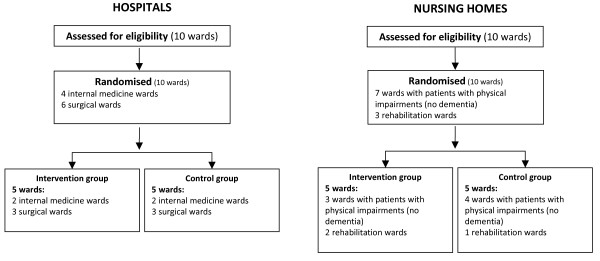
**Randomisation**.

A cluster randomised trial was conducted between September 2006 and November 2008 in the Netherlands. Hospitals and nursing homes were asked to participate with two or four, more or less comparable, wards. The hospital wards (n = 10) were internal medicine wards (n = 4) and surgical wards (n = 6) from four hospitals. The nursing home wards (n = 10) were wards with patients with physical impairments (no dementia)(n = 7) or rehabilitation wards (n = 3) from six nursing homes. The randomisation of the wards was stratified for centre and type of ward (Figure 1) and took place prior to baseline data collection.

Baseline data collection took place from September through November 2006. Subsequently, the patient safety program was implemented on the intervention wards: five hospital wards and five nursing home wards from December 2006 – February 2008. The wards of the control group continued care as usual. The follow-up period was nine months and continued until the end of November 2008.

The Medical Ethics Committee of district Arnhem – Nijmegen assessed the study and waived the need for complete evaluation of the study.

#### Study population

Adult patients (≥ 18 years) admitted to the hospitals or the nursing homes during our study, were asked to participate. Hospital patients with an expected stay of at least five days were asked within 48 hours after admission. After a written informed consent the research assistants visited the patients once a week. All patients with at least a second visit were included in this study.

All (clinical) nurses at the wards participated in our study. Nurses' aids and students were excluded.

#### Outcome measures

The primary outcome measure was the incidence of AEs (sum of the incidence of pressure ulcers, urinary tract infections and falls).

A *pressure ulcer *is an area of localised damage to the skin and underlying tissue caused by a combination of pressure and shear[21]. Pressure ulcers are classified in four grades according to the guidelines [19,21,40]. Pressure ulcers were considered present if a patient developed a PU grade 2 or worse. If a patient had a PU grade two or worse at the first visit, that PU lesion was excluded from the registration of PUs until the PU healed. Patients with an already present PU grade two or worse were only registered if they developed additional PU lesions.

A *urinary tract infection *is bacteriuria with clinical symptoms as: frequent urinating, pain while urinating, abdominal pain, fever and delirium, urinary incontinence [18,24]. During this study we defined a urinary tract infection as present if it was diagnosed by a medical doctor. Patients were excluded from the registration of urinary tract infection for a period of three weeks if they had a urinary tract infection until the infection was cured.

A *fall *is an unexpected event in which the participant comes to rest on the ground, floor, or lower level [20,41]. In this study the falls were measured by examining the patient files, assuming that if a patient fell it was reported in his or her file.

The secondary outcome measures were 1) the percentage of patients that received preventive care and 2) the knowledge of nurses regarding the three topics.

*Prevention *is important in patients at risk for one of the AEs. Preventive measurements were considered present when the care was performed according to the guideline.

The risk of *pressure ulcers *was measured with the PrePURSE [27] and the Braden scale [42] in hospitals and nursing homes, respectively. Next preventive care was measured: position while lying or sitting; if patients' heels were lifted; use of pressure-reducing material or alternating pressure material in bed or chair; presence of a repositioning scheme.

Hospital patients were at risk for a *urinary tract infection *if they had at least one of the next four risk factors [18,23]: 1) a urinary catheter in situ or the week before, 2) incontinence of faeces, 3) urinary retention or 4) a urinary tract infection in the last two years. According to the guideline, all nursing home patients were considered at risk for a urinary tract infection [18]. Next preventive care was measured: personal hygiene, frequent toilet visits, unnecessary indwelling catheter and unobstructed urine flow.

To identify hospital patients at risk for *falls *we used the STRATIFY [43]. According to the guideline all nursing home patients were considered at risk for falls, except those who were totally immobile [20]. Next preventive care was measured: if the file had a written multidisciplinary plan with multi-factorial preventive interventions; a periodic evaluation of the multidisciplinary plan; a periodic evaluation of the multi-factorial risk factors for falls.

The *knowledge *of nurses about risk assessment and effective preventive care was measured using a written knowledge test. Each topic had twenty questions, on which nurses could answer 'correct', 'not correct', or 'do not know'.

The knowledge test was developed from questionnaires [44] (knowledge test used in an implementation study of a pressure ulcer guideline in the Netherlands (Schoonhoven, L. 2004) and geriatric educational material of the prevention of falls, 2007) and student tests of the HAN University of Applied Sciences on the three topics. The face validity was tested by sending the questionnaire to the members of the research group (LS, JAJM, RTCMK and TvA), and the expert on each topic. Finally, nurses in hospitals and nursing homes were asked to pretest the questionnaire.

#### Data collection

During the baseline and follow up period, the *patient data *were collected in two ways. To measure AEs and preventive care the research assistants read the patient files and observed the patients during a weekly visit. To measure the utilization of preventive care, wards were visited three times by research assistants. At each visit they observed a sample of at least five patients and nurses during their daily activities for five hours.

All nurses were asked to fill in a questionnaire at the start of the baseline period and the follow-up period.

#### Statistics

Power calculation was based on the primary outcome, with a two-sided alpha of 0.05 and 80% power for the analysis of both the hospital and the nursing homes data.

As randomisation was on ward level, a ward was considered to be a cluster. To account for these clusters an intra class correlation coefficient of 0.01 was used in the calculation.

In hospitals, the incidence of pressure ulcers (10%) will be the highest contributor to our combined AE measure. The incidence of urinary tract infection and falls in the same patients is unknown. Therefore we assumed that the count of these three AEs will be 12% (an additional 1% for falls and 1% for urinary tract infections). We aimed to achieve a reduction of 50% as studies on the prevention of pressure ulcers have shown this is attainable [45,46]. To detect a decrease in AEs (from 12% – 6%) 1250 patients had to be included in each hospital group.

In the nursing homes, the incidence of falls will be the highest (60%). We assume that the additional contribution of pressure ulcers and urinary tract infection to AEs will be negligible. We aimed to achieve a reduction of 60% as a study on the prevention of falls showed this was attainable [47]. Therefore this study wanted to achieve a reduction of AEs from 60 – 36%. To detect this decrease in the nursing homes, 100 patients had to be included in each group.

The results will be analysed separately for hospitals and nursing homes, as patient characteristics, length of stay and nurse characteristics differ between hospitals and nursing homes.

The difference in incidence of AEs between the intervention and the control group during the follow up period will be analysed using a random effects Poisson regression analysis, including the following covariates: ward (random effect), institution and the baseline results of the ward.

The secondary outcomes will be evaluated in a similar way, using linear and logistic random effect models.

## Discussion

As implementation of a guideline is time-consuming, it is difficult for organisations to implement all available guidelines. Also, lack of feedback about performance using quality indicators of guideline based care and lack of a recognisable, unambiguous system for implementation often impede guideline implementation. A program that allows organisations to implement multiple guidelines simultaneously may facilitate guideline use and thus improve patient safety.

This study posed several challenges concerning the development of the complex intervention, the implementation of this intervention and the design of the trial. For the development of our intervention we used available guidelines on each topic. Translating three extensive guidelines into a manageable proposal for improving patient care is not easy. We chose to combine the essence of each guideline into a recognizable simple structural approach, and reduced the guidelines on each topic into two or three bundles. These bundles were easier to use in daily practice. The aim of the digital registration and feedback system was to provide the nurses on the ward with feedback on the performance of guideline based care. As we anticipated that nurses have limited computer skills and limited time to register all patients daily, we paid extra attention to the accessibility and performance of the digital program. This program was subsequently pre-tested during the first phase of this study in a group of future users and it was obvious that we had managed to develop a digital registration and feedback system that was user-friendly for all nurses on the wards. Also, the time it takes to register all patients on the wards was considered acceptable.

Our next challenge was the implementation of our intervention. Many factors may enhance or inhibit implementation. Therefore it is important to analyse the target group [16]. To be successful, we developed a multifaceted implementation strategy that could be tailored to each specific ward. By tailoring the strategy to the barriers of the individual wards we developed an individual implementation plan for each ward that considered the context of that particular ward.

The implementation of the digital registration and feedback system was even more complex. Currently, registration of patient care in a computer is not a standard procedure in the Netherlands. The nursing files are still mainly paper files. Moreover, not all nurses of the participating wards were used to working with a computer and on some wards the nurses did not even have access to a computer or the internet. We explored these barriers in a very early stage of the implementation process. This allowed us to remove the practical barriers, i.e. attaining access to a computer and the internet, and organise training programs for nurses to improve computer skills. Also, it gave the wards the opportunity to adopt the idea of registration of patient care on a computer. By the time they had to work with the digital registration and feedback system they were already used to the idea of using a computer.

Unfortunately it was not possible to prevent double registration of patient data: nurses had to write patient files and also register the patient daily care in the computer. This is only worthwhile when the digital program is of benefit to the nurses. Therefore, nurses were trained and encouraged to use the feedback provided by the digital program to evaluate and adjust daily care.

The final challenge we want to discuss is the design of the cluster randomised trial. Cluster randomised trials are more complex to perform, as they require more participants [48], due to the correlation between individuals in the same ward. In this study we took this into account by including an intra cluster correlation coefficient in the power calculation. As a result we had to include many hospital patients: 1250 in each group. To include and follow up that many patients in such a short time is ambitious, but we are convinced that it is achievable. Also, analyses of cluster randomised trials are complex. For analysing the effect of an intervention, a regression analysis including covariates should be used to account for the influence of the wards. Therefore this study will consider the following covariates: ward (random effect), institution and the baseline results of the ward.

Dissemination of the results of this study is planned for 2009.

## Competing interests

The authors declare that they have no competing interests.

## Authors' contributions

BGIvG wrote the article, developed the patient safety program, coordinated the study, prepared instruments for the study, and collected and analysed data. LS wrote the article, has been involved in the development of the patient safety program, preparing the instruments for the study, is the supervisor of the study. MEJLH had an important input during the development of the implementation strategies and tailoring of het implementation plan and in revising the article. JAJM has been involved in the development of the patient safety program and in revising the article. GFB is the statistician and has been involved in the design of the study. He performed the power calculation and the sample size considerations. RTCMK has been involved in the development of the patient safety program and in revising the article. TvA has been involved in the development of the patient safety program, preparing the instruments for the study, is the general supervisor of the study and in revising the article.

All authors approved the final version of the manuscript.

## Pre-publication history

The pre-publication history for this paper can be accessed here:


